# What matters in the annuitization decision?

**DOI:** 10.1186/s41937-022-00094-4

**Published:** 2022-06-21

**Authors:** Mohamad Hassan Abou Daya, Carole Bernard

**Affiliations:** 1 Department of Accounting, Law and Finance, Grenoble Ecole de Management, 12, Rue Pierre Sémard, 38000 Grenoble, France; 2 Faculty of Economics at Vrije Universiteit Brussel, Brussels, Belgium

**Keywords:** Annuities demand, Trust in financial analysts, Multiple factors test, D12, D83, G11, J14, J26

## Abstract

We perform a simultaneous test for several rational and behavioral factors known to affect the uptake of life annuities in a sample of Americans. We also investigate whether analysts’ short-term stock market expectations affect the decision to annuitize retirement wealth. We find that facing such expectations without trusting them lowers the purchase of annuities. Moreover, we find that individuals who trusted financial analysts’ expectations were less likely to purchase annuities. We attribute these findings to the availability heuristic and present bias, respectively. Finally, we discuss the mediating role of annuity antipathy. Our results provide guidance for policy-makers and annuity providers and offer venues for future research.

## Introduction

After Yaari's (1965) seminal paper, a vast and rich literature developed models providing evidence that rational individuals of retirement age should allocate a substantial portion of their wealth to life annuities. The main argument was that life annuities acted as an insurance policy for individuals against outliving their accumulated financial wealth because these investments provide income until death. This insurance is very valuable under the standard life-cycle model. Specifically, according to Yaari’s model, a` rational consumer should invest 100% of her wealth in life annuities to maximize her expected lifetime utility (frictionless market, no bequest motive). Surprisingly, the market for private life annuities is quite small. Therefore, economists use the term “annuity puzzle” to refer to this inconsistency between theory and observation.

A number of studies proposed rational and behavioral explanations in attempts to solve this puzzle. Researchers initially relied on the rational paradigm to propose factors, such as adverse selection (Finkelstein & Poterba, 2004) and bequest motives (Lockwood, 2012). Behavioral economists recently presented possible behavioral factors that may affect annuitization decisions. For example, Benartzi et al. (2011) noted that the inconsistency between theory and the market for annuities may be due to poor financial decision-making resulting from financial illiteracy. However, most prior studies individually tested each factor. In contrast, the present paper simultaneously tested several competing rational and behavioral explanations, which allowed the investigation of the relative importance of each factor. The result of this study should allow policy-makers to have a more informed prioritization of actions and alleviate the low demand for life annuities.

This paper provides three main contributions. First, we performed a simultaneous test of several factors that were hypothesized to affect the annuitization decision using an American sample. Prior literature primarily tested one factor at a time. To the best of our knowledge, only Goedde-Menke et al. (2014) tested various factors using a German sample. Most of their sample was comprised of individuals who were under 50 years of age and over 16 years of age. This sample is likely outside the annuity market, and their estimated effects may not reflect the population of interest. Using a more appropriate sample, we provide evidence that investment framing, self-selection (life expectancy), annuity literacy, family risk pooling, mortality salience and liquidity concerns jointly affect the annuitization decision. Notably, we provide an order for these effects that informs policy-makers. Second, we illustrate the mediating role of annuity antipathy in the uptake of life annuities. Third, we demonstrate the role of trust in financial analysts’ stock market expectations (TRUST hereinafter) in the uptake of life annuities. Previous literature primarily argued that distrust in financial institutions decreased the uptake of life annuities. In contrast, we provide evidence that TRUST has a negative relationship with the uptake of life annuities. We attribute our finding to present bias and the availability heuristic (Tversky & Kahneman, 1973).^1^ These results highlight the importance of specifying the division of financial institutions that individuals are trusting.

The remainder of this paper is organized as follows. Section 2 reviews the various factors affecting the decision to annuitize retirement savings. Section 3 describes the empirical methods used in this paper in the context of our study, and the results are presented in Sect. 4. Section 5 addresses important limitations. Section 6 provides the conclusion of the paper.

## Annuity puzzle explanations

Selection of the most important and relevant explanations for the annuity puzzle was based on the review papers of Alexandrova and Gatzert (2019) and Lambregts and Schut (2020) and our own research. The following sections present the rational and behavioral factors affecting the annuitization decision.

### Rational factors

Multiple scholars attempted to solve the annuity puzzle using rational factors. We present four important rational factors. The first factor is bequests. It is not optimal for individuals to annuitize their wealth if they want to leave money to their families (see Davidoff et al., 2005; Hainaut & Devolder, 2006; Purcal & Piggott, 2008; among others). However, empirical research mostly showed that the bequest motive had no average marginal effect on the annuitization decision (Bernheim, 1991; Brown, 2001; Hagen, 2015; Inkmann et al., 2011). However, this result does not suggest that bequest does not influence the annuitization decision based on individual income levels. For example, the bequest motive is relevant for wealthy individuals (Hurd, 1987) because bequest is essentially a luxury good. Therefore, we considered the bequest motive in our analysis and tested its interaction with the level of household income.

The second factor is having family members whom individuals can rely on to support them financially in times of need (Kotlikoff & Spivak, 1981). For example, if individuals know that they can count on their children to help them in old age, then they have no worries about outliving their wealth. To the best of our knowledge, only Goedde-Menke et al. (2014) (**GM** hereinafter) empirically tested this notion and found results in support of it.

The third factor is subjective perception of life expectancy, or more generally, adverse selection. People who think that they may have shorter lives than their peers should in general choose to annuitize less of their retirement wealth. Empirical research mostly provided evidence to support this notion (Guillemette et al., 2016; Hagen, 2015). However, **GM** found an opposing effect of subjective life expectancy on the decision to annuitize retirement wealth but offered no explanation of this finding.

The fourth factor is the loss of liquidity. Individuals lose the ability to cover urgent expenses or invest in very attractive opportunities when choosing to buy an annuity. The few available empirical studies that specifically tested this factor provided evidence that loss of liquidity had a negative effect on the annuitization decision (Beshears et al., 2014; Chalmers & Reuter, 2012) or no effect (Goedde-Menke et al., 2014).

### Behavioral factors

This section presents five important behavioral factors affecting the annuitization decision. The first and most prominent behavioral explanation of the annuity puzzle is the framing effect (Agnew et al., 2008; Brown et al., 2008; Gazzale et al., 2012; Nolte & Langer, 2016). Framing the annuity in terms of an investment rather than in consumption opportunities may lead individuals to consider the annuity as a gamble on their life rather than an insurance against outliving their wealth (Brown & Warshawsky, 2004). Therefore, individuals prefer life annuities over savings accounts when annuities are framed in consumption terms (Brown et al., 2008). Framing the life annuity as an investment places it in a mental account, which triggers loss aversion and leads to its undervaluation (Hu & Scott, 2007).

The second behavioral factor affecting life annuity uptake is that the idea of annuitization triggers thoughts related to death. Therefore, people tend to avoid annuitizing their wealth as a type of defense against these thoughts (Salisbury & Nenkov, 2016). We expect that this type of defense works at the implicit unconscious level rather than the explicit conscious level. We tested this notion by checking the mediation of the explicit attitude toward annuities.

The third factor affecting the annuitization decision is time preference. The fact that annuities shift the receipt of income from the present to the future makes it undesirable due to hyperbolic discounting (Hu & Scott, 2007). Consistent with this notion, Schreiber and Weber (2016) provided evidence that individuals who were of retirement age were more likely to choose to receive their pension savings as a lump sum compared to younger individuals. Therefore, we included the subjective discount rates of individuals in our study.

The fourth behavioral factor affecting the low uptake of life annuities is the absence of the proper cognitive ability to value them. Recent literature discussed the effects of financial literacy on the uptake of life annuities. The literature provided evidence that people with poor financial literacy were less likely to plan their retirement or accumulate retirement wealth (Lusardi & Mitchell, 2007, 2011), and most households exhibited poor financial literacy (Lusardi, 2019; Lusardi & Mitchell, 2014). Therefore, it is not surprising that the private annuity market is small. However, the literature provides competing points of view. For example, **GM** provides evidence that a more specific annuity literacy measure is related to the low uptake of life annuities. When controlling for annuity literacy, they found that financial literacy was negatively related to the uptake of life annuities. Therefore, we included both measures for both literacy types in our study.

The fifth behavioral factor affecting the annuitization decision is the availability heuristic. Prior literature provided evidence that the valence of previous experiences with annuities had a positive relationship with life annuity uptake (Agnew et al., 2015). However, it is unlikely that an individual buys an annuity twice in their lifetime. We present an alternative mechanism of how the availability heuristic affects the annuitization decision. We posited that when individuals were faced with expectations of stock market movements, they recalled alternative methods to finance their annuity, such as stocks and bonds. It is easier to imagine situations where stocks and bonds may be better. However, this effect would depend on how much the individuals trusted the analyst presenting the expectations. We initially expected that there would be a negative relationship between this trust and the uptake of annuities. Our expectation was based on prior findings of a negative relationship between recent stock market movements and the uptake of life annuities (Chalmers & Reuter, 2012; Previtero, 2014).

### Mediating role of attitude

Many studies presented convincing factors that affect the annuitization decision. However, few of these studies explained the channels through which each factor affected the decision. The present study delineated which factors affected the annuitization decision via the channel of explicit attitude toward the annuity as a financial or insurance product. Shu et al. (2016) showed that between 15 and 20% of their sample decided to self-manage their retirement wealth even when the expected payout of the annuity was more than double the down payment. They called that portion of their sample “annuity haters”. Therefore, it is important to test which factors affect the annuitization decision using the channel of explicit attitude toward annuities, and we included a measure of annuity antipathy in our study to test its mediating role for all our factors.

## Empirical methods

### Survey design

After running a pretest with 310 individuals, we performed our survey on May 6, 2020. We used Prolific platform to recruit 720 Americans who were at least 50 years old and resided in the USA at the time of the study. We used the Prolific platform because, unlike in Mturk, the participants were recruited for research purposes only (Palan & Schitter, 2018). We chose individuals who were at least near retirement because asking young individuals about retirement decisions is not relevant in solving the annuity puzzle.

The participants were randomly assigned to one of three conditions. Participants in the control condition answered the following hypothetical scenario: “Suppose that you are 65 years old. You are about to retire. You have saved $100,000 in your pension plan. Please select one of the two options below regarding your pension plan”. We added one of these statements in the other two conditions: “Financial analysts expect that the stock market will rise this year” (UP condition hereafter) or “Financial analysts expect that the stock market will fall this year” (DOWN condition hereafter). The respondents chose between receiving their retirement savings as a lump sum upfront or $650 monthly for the rest of their lives in all scenarios. Before presenting the hypothetical scenarios, we provided a definition for the annuity that we retrieved from the low-mortality salience case of Salisbury and Nenkov (2016). To prevent missing data, the individuals had to respond to all our questions.

The respondents took 9 min on average to complete the survey. Therefore, we do not expect that our respondents had overloading effects in completing the survey. Thirty-seven of the 720 individuals did not pass our attention check, and seven reported ages younger than 50 years. These individuals were excluded from our analyses. Therefore, we obtained 222 participants in the DOWN condition, 225 participants in the UP condition, and 229 participants in the control condition. Table 1 presents the characteristics of our sample.

**Table 1:** Sample characteristics

Variable	Mean	US population mean (%)
Sample size	676	
Gender		
Female	60.1%	50.9
Male	39.9%	49.1
Age		
50–54	28.1%	20.2
55–64	48.2%	39.2
65–80	23.7%	40.6
Education		
< High school degree	0.1%	10.91
High school graduate	8.7%	30.69
Some college but no degree	20.7%	15.11
Associate degree	10.5%	10.11
Bachelor's degree	36.7%	19.78
Master's degree	17.5%	9.65
Doctoral degree	2.5%	2.22
Professional degree	3.3%	1.53
Marital status		
Married	55.0%	49.4
Single	45.0%	50.6
Household income		
< $10,000	3.0%	5.47
$10,000–$19,999	9.5%	8.24
$20,000–$29,999	11.1%	8.21
$30,000–$39,999	11.1%	8.09
$40,000–$49,999	9.2%	7.44
$50,000–$59,999	7.7%	7.23
$60,000–$69,999	8.7%	6.25
$70,000–$79,999	8.3%	5.89
$80,000–$89,999	5.2%	5.01
$90,000–$99,999	4.3%	4.43
$100,000–$149,999	13.5%	13.40
$150,000+	8.6%	13.43

This table presents the distribution of our sample according to gender, age, education, marital status and household income. US population means are based on the Current Population Survey 2021

Table 1 shows that our sample was quite different than **GM**. Our sample had more individuals with at least a bachelor’s degree, a higher percentage of females, and higher household income levels.^2^ These differences were expected based on the general differences between the US and German populations and because our sample was comprised of individuals who were at least 50 years of age. As previously noted, most of the **GM** sample was comprised of individuals who were younger than 50 years of age but older than 16 years of age. Their estimated effects may not reflect the population of interest because the bulk of their sample was outside the annuity market. Table 1 also shows that our sample is close in its income distribution, age distribution and marital status to the US population. It is good to note that getting a completely representative sample for a specific age group is not an option on the Prolific platform.

### Measuring trust in financial analysts’ expectations

We measured TRUST using a three-item 7-point Likert scale anchored by 1 (strongly disagree) and 7 (strongly agree).^3^ The three items were “Financial analysts' stock market expectations are trustworthy,” “I could rely on financial analysts' stock market expectations,” and “Financial analysts' stock market expectations are mostly accurate”. We pretested these measures using 30 individuals. This measure had a Cronbach’s alpha of 0.9 and a KMO of 0.75. These results indicated that the measure was internally consistent and valid.

### Measuring annuity possession motives

We measured family risk pooling, self-selection, investment frame, and liquidity concern using the items proposed by **GM**. We adapted the bequest motive measure from the same paper to “I want to leave money for my family after I die” because we were not certain that everyone would understand the meaning of “Bequeath”.

We measured annuity antipathy and mortality salience using the level of agreement with the statements “I dislike annuities” and “I had death-related thoughts while answering the hypothetical scenario question,” respectively. We also used a 7-point Likert scale anchored by 1 (strongly disagree) and 7 (strongly agree) for both measures.

### Measuring financial and annuity literacy

We measured financial literacy using the items suggested by Lusardi and Mitchell (2011) and an additional 3 questions of the advanced financial literacy items that were suggested by Van Rooij et al. (2011). We added three additional questions to ensure variability in our financial literacy measure.

We also measured basic annuity literacy using the items proposed by **GM**.

### Control variables

We included questions in our survey to elicit/measure loss aversion (adapted from Tversky & Kahneman, 1992), risk aversion, subjective discount rate, household income, age, gender, level of education, and marital status.

We measured loss aversion by asking the participants to select one of nine ranges, which included the minimum amount of winning with a 50% chance for which they would be willing to participate in a lottery in which they had a 50% chance of losing $25, $50 and $75. We used the middle value of each range to calculate loss aversion. We calculated a Cronbach’s alpha of 0.97 and a KMO of 0.74 for our loss aversion measure.

We measured the subjective discount rate by asking the participants to provide the amount of money to be received in 1 month, 6 months, and 1 year, which would make it equivalent to receiving 100 US dollars upfront. Therefore, we obtained three discount rates, which we averaged to obtain our time preference measure. We calculated a Cronbach’s alpha of 0.9 and a KMO of 0.7 for our time preference measure. Therefore, it seemed that our participants had a constant subjective discount rate.

We also included the duration that each participant took in answering the hypothetical scenario question as a control variable. We included this variable to control for whether individuals selected the answer in a more random manner when answering relatively quickly.

All other control variables were shown to affect the annuitization decision in theoretical or empirical studies.

## Results

### Summary statistics

Table 2 reports the summary statistics of our non-demographic continuous variables. Table 2 shows that elderly individuals are, on average, risk averse, have high subjective discount rates, highly loss averse and have good annuity and financial literacies. It is notable that 36% of our sample decided to annuitize their wealth, which is comparable with **GM**. The latter had a 17.5% annuitization rate while having 46% of the sample with an age of 44 years or less. It is notable that the annuitization rate for the control, DOWN and UP Groups was 36%, 34%, and 38%, respectively. Consequently, overall it does not seem that the experimental conditions affect the annuitization decision without taking into consideration the moderation of the trust in financial analysts.

**Table 2:** Summary statistics

Variable	Mean	SD	Min	Max
Annuity	0.36		0	1
TRUST	3.96	1.10	1.67	7
Basic annuity literacy	2.27	0.91	0	3
Financial literacy	3.78	1.12	0	5
Loss aversion	3.54	2.71	0.5	8
Time preference	0.50	0.34	0.05	0.8
Risk aversion	0.66	0.35	0.29	1.15

This table displays the mean, standard deviation, the minimum value and the maximum value of each listed variable

Table 3 displays the distribution of answers on the Likert scales of the various motives questions. From Table 3, it is clear that individuals tend to have liquidity concerns, view the annuity in an investment frame, and have a bequest motive.

**Table 3:** Distribution of motive answers

Motive	Answers to Likert scale (1 to 7) (%)						
	1	2	3	4	5	6	7
Liquidity	2.22	7.99	14.69	9.32	27.66	23.22	14.94
Family risk pooling	14.79	17.75	14.05	15.53	23.52	10.5	3.85
Bequest	9.91	9.32	9.62	10.5	18.49	25.44	16.72
Investment frame	0.0	2.51	2.07	6.8	23.52	38.91	26.18
Mortality salience	26.33	18.79	6.8	5.62	18.34	16.57	7.54
Self-selection	1.48	5.18	12.13	40.09	17.9	17.31	5.92

This table displays the distribution of answers of 676 survey participants on the motives questions

### Manipulation check

We checked manipulation in our experiment by asking the participants to rate their level of agreement with the statement, "In the hypothetical scenario, I think that a group of financial experts would expect the stock market to rise this year", anchored by 1 (strongly agree) and 7 (strongly disagree). Please note that the manipulation questions were asked after the decision to purchase the annuity question to prevent possible priming effects. We found that individuals in the UP condition had a higher level of agreement (*M* = 2.12, SD = 1.33) with the aforementioned statement than individuals in the control condition (*M* = 3.79, SD = 1.57; *t* (673) = 11.63, *p* < 0.01) and the DOWN condition (*M* = 5.35, SD = 1.67; *t* (673) = 22.3, *p* < 0.01). Therefore, our manipulation was successful.^4^

### Simultaneous test result

We ran two logistic regressions to investigate the effects of the various factors, as shown in Table 4. Regression (1) in Table 4 displays the results of our experimental factors without literacy and control measures. Regression (2) displays the marginal log-odds effects of all factors for the refined sample of 676 participants. Please refer to “Appendix 1” for a description of all variables. The dependent variable in both regressions had a value of zero for individuals who selected the lump sum option and a value of one for the individuals who selected the annuity option.

**Table 4:** Logistic model results

Variables	Selected annuity = Yes	
	(1)	(2)
*Experiment*		
UP	− 1.589**	− 2.272**
	(0.745)	(0.924)
DOWN	− 1.425*	− 1.850*
	(0.773)	(0.972)
TRUST	− 0.495***	− 0.554***
	(0.134)	(0.171)
UP:TRUST	0.415**	0.559**
	(0.185)	(0.230)
DOWN:TRUST	0.323*	0.402*
	(0.192)	(0.239)
*Motives*		
Liquidity		− 0.438***
		(0.067)
Family risk pooling		− 0.125**
		(0.061)
Bequest		0.186**
		(0.094)
Bequest:income		− 0.215*
		(0.117)
Investment frame		− 0.413***
		(0.094)
Mortality salience		− 0.087*
		(0.048)
Self-selection		0.172**
		(0.076)
*Literacy*		
Basic annuity literacy		0.598***
		(0.122)
Financial literacy		− 0.187*
		(0.096)
*Controls*		
Further controls	NO	YES
Observations	676	676
Wald-Chi^2^	15.6***	116.0***
Pseudo R^2^	0.019	0.197

This table displays the log-odds effects of all our variables

The dependent variable is whether the individual selected the annuity in the hypothetical scenario question. Robust standard errors in parentheses. ***p < 0.01, **p < 0.05, *p < 0.1

We tested our independent variables for multicollinearity issues using the variance inflation factor (VIF), although it is not perfectly suitable for logistic regressions. Our range of VIFs was 1.0 to 1.6. Therefore, we did not seem to have multicollinearity problems in our regressions.

We probed the interaction effects of UP and TRUST and DOWN and TRUST using the Johnson-Neyman technique (Johnson & Fay, 1950) in regression (1). We found that when TRUST was below 2.93, the marginal effect of the UP condition was negative and significant at the 5% level. We found that when TRUST was below 1.19, the marginal effect of the DOWN condition was negative and significant at the 5% level. These results suggests that the availability heuristic possibly affected the annuitization decision, as expected. However, we did not find a negative relationship between the direction of the expected market movement and TRUST. Because many factors are at play, such as expectations of economic conditions and default probabilities, we did not attempt to explain the interaction. However, TRUST was the most important variable overall. Intuitively, individuals can gain money within a year based on the expectations of stock market movements regardless of the direction. Individuals can short or long the market to obtain gains. Even if the individuals have difficulties in shorting the market, they can long financial instruments such as inverse exchange traded funds which track the market. The price of such funds moves in almost perfect opposition to the market. Therefore, one possible explanation to the observed relationship between TRUST and annuity uptake is that due to present bias, individuals who trusted the expectations of short-term market movements would more likely take their retirement savings in cash rather than as an annuity. One could argue that an alternative explanation would be that experienced investors trust financial analysts more and are—due to their experience with investing—less likely to buy annuities. We leave such an alternative explanation for future research although the increased “experience with investing” could mean that the individuals have higher availability for scenarios where annuities are not optimal for their retirement.

Regression (2) shows that all factors had effects which were consistent with prior empirical findings. Liquidity concerns, family risk pooling, investment framing, mortality salience, and basic annuity literacy reduced the likelihood that individuals selected the annuity options, but self-selection (life expectancy) increased it. The interaction between bequest motives and household income was significant at the 5% level. However, a Johnson-Neyman analysis of the interaction showed that household income should be less than $11,000 (approximately 3% of our sample) for the bequest motive to have a significant negative effect on the annuitization decision. Therefore, we concluded that bequest motives had no effect on the annuitization decision.

To obtain a better picture of the role each factor played in the annuitization decision, we computed the average marginal effects (AME hereinafter). We report our AME and the AME of **GM**^5^ in Table 5 for comparison purposes. We found that AME of liquidity (7.7 pp) and investment frame (7.2 pp) almost tied for first place. The AME of TRUST (4.1 pp) was in second place, the AME of self-selection (3 pp) came in third place, the AME of family risk pooling (2.2 pp) came in fourth place, and the AME of mortality salience (1.5 pp) came in fifth place. The AME of bequest was not significant. These results are consistent with our previous finding.

**Table 5:** Average marginal effects

Variables	This study	GM
*Experiment*		
UP	− 0.021	
	(0.040)	
DOWN	− 0.053	
	(0.040)	
TRUST	− 0.041***	
	(0.016)	
*Motives*		
Liquidity	− 0.077***	0.004
	(0.010)	(0.025)
Family risk pooling	− 0.022**	0.017
	(0.010)	(0.025)
Bequest	0.008	− 0.058**
	(0.009)	(0.023)
Investment frame	− 0.072***	− 0.079***
	(0.015)	(0.027)
Mortality salience	− 0.015*	
	(0.008)	
Self-selection	0.03**	− 0.085***
	(0.013)	(0.027)
*Literacy*		
Basic annuity literacy	0.105***	0.113***
	(0.020)	(0.028)
Financial literacy	− 0.033**	− 0.106***
	(0.017)	(0.037)
Observations	676	1394

This table presents the average marginal effects of the factors of the regressions in Table 2. Delta-method standard errors in parentheses. ***p < 0.01, **p < 0.05, *p < 0.1

Comparisons of our AME values to GM revealed several interesting findings, some of which highlight the differences between the German and American populations. First, in contrast to our results, **GM** did not have a significant effect for liquidity concerns. One simple explanation is that Germans can rely on the healthcare, social security systems, and the obligations of their children to cover up health and living expenses. Second, the AME of self-selection was negative in **GM**, which suggests that people who expect to live longer do not annuitize their wealth. Although this is surprising, it might be the case that Germans who believe they will live longer than their peers boast better health expectations. Therefore, they can stay in the workforce even after official retirement. However, this should be tested in future studies. Third, **GM** had a significant effect for bequest motives. This result may be driven by general differences between German and American populations. In contrast to European countries, the USA lacks stringent inheritance rules (Alesina & Giuliano, 2014). Therefore, an individual with a bequest motive will not necessarily follow through with the bequest in the USA, and the bequest motive did not play a role in the annuitization decision in the USA. Fourth, the AME values for annuity literacy and investment framing were very close in both studies. This result suggests that the effects of those two factors may be generalized beyond the boundaries of either nation. Fifth, financial literacy measures were different in both studies, and their AME values were not comparable in magnitude. However, the AME values of financial literacy had the same sign in both studies and were significant at the 5% level.

### Identification of the effects mediated by explicit attitudes toward annuities

To determine which effects were mediated by the participant’s attitude toward the annuity, we ran a “mediation analysis using process” (Hayes and Rockwood 2020). Table 6 presents the direct and indirect effects of motives and literacy measures on the annuitization decision. Out of all the motives, only the effects of liquidity and investment framing were mediated by disliking annuities while the effects of both annuity and financial literacy were mediated by disliking annuities. In what follows, we discuss the direct and indirect effects of each variable. All of the effects discussed in the following discussion refer to the effect on the annuitization decision.

**Table 6:** Direct and indirect effects of motives and literacy

Dependent variable:	Selected annuity = Yes	
Variables	Direct effect	Indirect effect
*Motives*		
Liquidity	− 0.357***	− 0.128**
	(0.061)	(− 0.193, − 0.074)
Family risk pooling	− 0.123**	− 0.173
	(0.053)	(− .064, 0.030)
Bequest	− 0.015	− 0.005
	(0.047)	(− 0.046, 0.034)
Investment frame	− 0.370***	− 0.138**
	(0.084)	(− 0.214, − 0.072)
Mortality salience	− 0.100**	− 0.023
	(0.043)	(− 0.060, 0.011)
Self-selection	0.228***	0.011
	(0.072)	(− 0.050, 0.073)
*Literacy*		
Basic annuity literacy	0.279***	0.277**
	(0.108)	(0.195, 0.379)
Financial literacy	0.014	− 0.120**
	(0.079)	(− 0.205, − 0.048)

This table presents the log-odds direct and indirect effects of the motive and literacy variables. The mediator is taken as annuity antipathy (dislike of annuities). The dependent variable is whether the individual selected the annuity in the hypothetical scenario question. Robust standard errors and 95% CI in parentheses for direct and indirect effects. Effects are expressed in log-odds. ***p < 0.01, **p < 0.05, *p < 0.1

First, liquidity had significant negative direct and indirect effects. When individuals wanted to keep their savings available at all times, they viewed annuities unfavorably. These individuals would not buy an annuity because they had a need for savings to pay for medical costs, long-term care, or lucrative investment opportunities.

Second, family risk pooling only had a significant negative direct effect. This result was reasonable because there was no logical relationship between having the ability to rely financially on family members and disliking the annuity as a product.

Third, bequest did not have a significant effect, which is consistent with our previous findings.

Fourth, investment framing had significant negative direct and indirect effects. Viewing the annuity as an investment affected the attitude toward it because overall annuities have low returns. Viewing annuities as an investment would reduce the need for individuals to optimize consumption after retirement because alternative methods would yield better returns.

Fifth, mortality salience had a significant negative direct effect only. This result provides evidence that mortality salience works at a subconscious level because the explicit attitude toward annuities did not mediate the effect of mortality salience. However, this notion should be further tested in a high mortality salience case. An implicit association test would be very interesting for future research.

Sixth, self-selection had a significant positive direct effect only. This result showed that self-selection only affected the annuitization decision via the need for an annuity to optimize utility from consumption after retirement.

Seventh, annuity literacy had significant positive direct and indirect effects. These results showed the importance of annuity literacy in lowering hatred toward annuities and increasing annuity adoption via multiple channels. Learning more about annuities would definitely decrease hatred toward them and boost the need for annuities to provide insurance against outliving ones’ wealth.

Eighth, financial literacy only had a significant indirect effect. Therefore, we primarily found that financially literate people tended to dislike annuities. However, the reasons behind this dislike are still not clear-cut and would be interesting for further research.

## Robustness checks

### Median-split motives

In regression (2) of Table 4, the motive variables follow an ordinal scale. To check whether the motive effects still hold in a more appropriate statistical setting, we convert the motive variables into binary variables having a value of one, if the value of the motive variable is above the median, and zero otherwise. Table 7 displays the average marginal effects after median-splitting the motive variables. Overall, the motive variables retain their significant effects in the same direction. Mortality salience becomes significant at the 1% level, and self-selection becomes significant at near 5% level (*p* = 0.056). Therefore, the results are robust to median-splitting the motives variables.

**Table 7:** Average marginal effects with median-split motives variables

Variables	Selected annuity = Yes
*Experiment*	
UP	− 0.004
	(0.041)
DOWN	− 0.042
	(0.040)
TRUST	− 0.038***
	(0.016)
*Motives*	
Liquidity	− 0.236***
	(0.032)
Family risk pooling	− 0.080**
	(0.035)
Bequest	0.005
	(0.036)
Investment frame	− 0.140***
	(0.052)
Mortality salience	− 0.096***
	(0.035)
Self-selection	0.066*
	(0.034)
*Literacy*	
Basic annuity literacy	0.109***
	(0.020)
Financial literacy	− 0.024
	(0.017)
Observations	676
Wald-Chi^2^	136.47***
Pseudo R^2^	0.155

This table presents the average marginal effects of the factors of the regressions in Table 2 after median-splitting the motive variables. Delta-method standard errors in parentheses. ***p < 0.01, **p < 0.05, *p < 0.1

### Linear probability model

To qualitatively check for the robustness of the average marginal effects of our logit model in Table 5, we run a linear probability model. Table 8 displays the average marginal effects of both models. It seems that the effects are qualitatively robust to this model specification.

**Table 8:** Comparison of average marginal effects between logit model and LPM

Variables	Logit	LPM
*Experiment*		
UP	− 0.021	− 0.020
	(0.040)	(0.041)
DOWN	− 0.053	− 0.054
	(0.040)	(0.041)
TRUST	− 0.041***	− 0.034**
	(0.016)	(0.016)
*Motives*		
Liquidity	− 0.077***	− 0.080***
	(0.010)	(0.011)
Family risk pooling	− 0.022**	− 0.021**
	(0.010)	(0.011)
Bequest	0.008	0.006
	(0.009)	(0.010)
Investment frame	− 0.072***	− 0.072***
	(0.015)	(0.016)
Mortality salience	− 0.015*	− 0.011
	(0.008)	(0.008)
Self-selection	0.030**	0.032**
	(0.013)	(0.013)
*Literacy*		
Basic annuity literacy	0.105***	0.098***
	(0.020)	(0.018)
Financial literacy	− 0.033**	− 0.029*
	(0.017)	(0.017)
Observations	676	676
Pseudo R^2^	0.197	
R^2^		0.192

This table presents the average marginal effects of the factors of the regressions in Table 2 alongside the average marginal effects estimates from a linear probability model. Standard errors in parentheses. ***p < 0.01, **p < 0.05, *p < 0.1

### Supplementary survey

The previous survey had three main limitations that we must address. The first limitation is that we did not control for hypothetical bias beyond the use of the ex-ante technique of reminding the participants that there were no right or wrong answers. We assumed that the individuals would answer in a manner that reflected their true behavior. The second limitation is that we did not directly control for a competing hypothesis, which claims that trust in financial institutions is a driver of annuity uptake. The third limitation is that our scale of trust was not bounded by “not at all” and “completely.”

Therefore, we performed a smaller similar survey in which we addressed these three limitations. The new survey included 350 participants, and 10 failed our attention check. We only included the control condition of our first survey because our primary aim was to test the opposing trust hypotheses, which was our main finding. To address our first limitation, we controlled for hypothetical bias via the use of the uncertainty recording technique (see Samnaliev et al., 2006; Lundhede et al., 2009). To address our second limitation, we measured trust in financial institutions using a 7-point Likert scale for the item “To what extent do you trust financial institutions such as banks and insurers?”. To address the last limitation, we set the anchors of all trust scales to “not at all” and “completely”.

This study had results that were consistent with our previous study. For brevity, we only mention the results of the competing trust-based hypotheses. Trust in financial institutions had an insignificant AME of 1.4 percentage points (*p* > 0.1), and TRUST had a significant AME of -5.2 percentage points (*p* < 0.05). Notably, when TRUST was absent from the model, then trust in financial institutions had a significant positive impact on annuity uptake. This result reinforces the notion that researchers should be specific in specifying which financial institutions the individuals are trusting.

## Conclusions

The present paper simultaneously tested multiple hypotheses about the low uptake of life annuities in a sample of Americans. We provide evidence that investment framing, self-selection (life expectancy), annuity literacy, family risk pooling, mortality salience, and liquidity concerns jointly affected the annuitization decision. In contrast, we did not find empirical support for the hypothesis that bequest motives affected the annuitization decision. We also provide a ranking for the effects of motives.

Based on our empirical findings, annuity providers should present their annuities in an insurance framework and in a manner that induces low levels of mortality salience. They should also adapt their products to the liquidity needs of individuals. Policy-makers should focus on educating the population about annuities to increase annuity literacy and reduce the deep aversion toward annuities.

We also constructed a measurement of trust in financial analysts’ expectations, which we used to provide evidence that financial analysts’ expectations of market movements affected the tendency of individuals to annuitize their retirement wealth according to the levels of trust in these expectations. We attribute these findings to the availability heuristic and present bias.

Note that we conducted the survey after the beginning of the corona virus pandemic. Hence, our results may be affected by the uncertainty imposed by the event.

## Data Availability

The datasets used and/or analyzed during the current study are available from the corresponding author on reasonable request.

## Appendix 1: Variable descriptions

See Table

**Table 9:** Variable descriptions

Variable	Description
UP	An experimental condition where financial analysts expect the stock market to move upward this year
DOWN	An experimental condition where financial analysts expect the stock market to move downward this year
TRUST	Trust in financial analysts' expectations
UP:TRUST	Interaction of up and trust
DOWN:TRUST	Interaction of down and trust
Liquidity	Fear of losing liquidity
Family risk pooling	Having family members that individuals can rely on financially
Bequest	Wanting to leave money for family members after death
Bequest:income	Interaction of bequest and household income
Investment frame	Thinking of an annuity as an investment rather than an insurance
Annuity antipathy	Deep annuity aversion
Mortality salience	Increased accessibility of death-related thoughts
Self-selection	Life expectancy compared to peers
Further controls	Time spent on answering the hypothetical scenario question, loss aversion, risk aversion, subjective discount rate, education level, female, number of children, and household income

9.

## Abbreviations

AME: Average marginal effect
GM: Goedde-Menke, Lehmensiek-Starke, and Nolte 2014
ETF: Exchange traded fund

## Footnotes

1 The availability heuristic or availability bias is a mental shortcut that relies on immediate examples that come to a person’s mind when making a decision.

2 **GM** study has 21.2% of their sample having at least a bachelor degree, 51.1% of their sample who identify as female, and only 6.7% of their sample earn 42,000 euros per year (this is their max category).

3 We are well aware that it would have been more adequate to anchor the scale by “Not at all” and “Completely”. Therefore, we performed another survey in the robustness checks section to address this short-coming.

4 There was no interaction between TRUST and either condition in our manipulation check (*p* = 0.67 and *p* = 0.51 for the interaction of TRUST with UP and DOWN, respectively). TRUST was not significantly different across the three conditions* F* (2, 673) < 1.

5 The AME of **GM** is for the logistic regression in which individuals who did not know whether they had an annuity were not included.
